# Defining the clinical genomic landscape for real-world precision oncology

**DOI:** 10.1016/j.ygeno.2020.10.032

**Published:** 2020-11

**Authors:** Philip A. Beer, Susanna L. Cooke, David K. Chang, Andrew V. Biankin

**Affiliations:** a Sanger Institute, Wellcome Trust Genome Campus, Cambridge CB10 1SA, United Kingdom; b Wolfson Wohl Cancer Research Centre, Institute of Cancer Sciences, University of Glasgow, Garscube Estate, Switchback Road, Bearsden, Glasgow, Scotland G61 1QH, United Kingdom; c West of Scotland Pancreatic Unit, Glasgow Royal Infirmary, Glasgow G31 2ER, United Kingdom; d South Western Sydney Clinical School, Goulburn, St, Liverpool NSW, 2170, Australia

**Keywords:** Genonics, Cancer, Molecular profiling

## Abstract

Through the delivery of large international projects including ICGC and TCGA, knowledge of cancer genomics is reaching saturation point. Enabling this to improve patient outcomes now requires embedding comprehensive genomic profiling into routine oncology practice. Towards this goal, this study defined the biologically and clinically relevant genomic features of adult cancer through detailed curation and analysis of large genomic datasets, accumulated literature and biomarker-driven therapeutics in clinic and development. The characteristics and prevalence of these features were then interrogated in 2348 whole genome sequences, covering 21 solid tumour types, generated by the PCAWG project. This analysis highlights the predominant contribution of copy number alterations and identifies a critical role for disruptive structural variants in the inactivation of clinically important tumour suppressor genes, including PTEN and RB1, which are not currently captured by diagnostic assays. This study defines a set of essential genomic features for the characterisation of common adult cancers.

## Highlights

• Genomic profiling requires coverage of all variant classes to unlock full utility.
• Small variants comprise just under a half of all clinically useful information.
• Appropriate analysis of copy number and structural alteration is essential.
• Available assays are frequently suboptimal in gene and variant class selection.
• Comprehensive profiling is within the economic reach of socialised healthcare.

## 1 Introduction

Over the past decade, substantial knowledge has accumulated around the genomic aberrations that underpin the development and progression of cancer, through the concerted efforts of large worldwide collaborations including the International Cancer Genome Consortium (ICGC), The Cancer Genome Atlas (TCGA) and, most recently, the Pan-Cancer Analysis of Whole Genomes (PCAWG) [2,14,16]. As a consequence, the discovery of common/phenotypically strong cancer genes, which contribute the majority of driver events, is now close to saturation. Indeed, in a recent analysis of 2658 whole genomes, only around 5% of cases did not have their genomic driver(s) identified [16]. Our current level of knowledge, therefore, is sufficient to define the vast majority of the clinically and biologically relevant cancer genomic space with a high degree of certainty.

Whilst this lexicon of cancer variants is close to completion, genomic profiling has yet to deliver on its potential for improving outcomes for cancer patients [17]. Advances to date have largely been based on a single gene - single drug paradigm delivered through a limited genomic test (a ‘companion diagnostic’), as exemplified by EGFR inhibitor therapy for *EGFR-*mutant lung cancers. While this model achieves dramatic responses for some patients, there is growing appreciation that a different approach is required to unlock the full potential of precision medicine, in order to help a wider range of cancer patients and to prevent the often rapid acquisition of resistance to targeted therapies [3].

Precision oncology requires an integrated analysis of the full complement of genomic events that underpin malignant transformation, disease progression and therapeutic response. Achieving this in the real world requires an assay that is able to deliver high-quality information from available biopsy material, requiring tolerance for small samples, formalin-exposed DNA and low tumour cellularity [18,20]. In addition, this information needs to be deliverable at scale, at a cost and using infrastructure that is achievable within publicly funded healthcare systems. These technical specifications are not yet met by whole genome sequencing (WGS) and hence WGS has been commissioned for only 3% of cancer cases in England following completion of the 100,000 genomes project (www.england.nhs.uk/publication/national-genomic-test-directories/). Whole exome sequencing (WES) is often viewed as the second choice when WGS is not possible. However, WES is not a good option for cancer profiling; firstly, because the vast majority of genes are not cancer genes and therefore most of the sequencing data generated from WES has no clinical utility, and secondly, because cancers contain driver events out with the coding exome, including alterations in regulatory regions [11,27] and fusion gene junctions, that may be missed by WES assays. Targeted capture sequencing technology is able to deliver all classes of genomic variant, and deployment of this approach will ensure the majority of cancer patients benefit quickly from the genomic knowledge delivered by ICGC, TCGA and PCAWG.

A current critical bottleneck in the translation of accumulated genomic knowledge into improved outcomes for patients is the accurate definition of content for targeted cancer assays, such that the maximum amount of genomic information is delivered for the maximum number of patients. To address this challenge, we performed an objective and exhaustive curation of published studies and databases to define the genomic features that drive neoplastic transformation, disease course, and therapeutic response and resistance. The prevalence in cancers and the type of genomic events affecting these features were then characterised in detail using 2348 cancer whole genome sequences from the PCAWG dataset. These data inform the design of cancer assays able to deliver the vast majority of clinically and biologically relevant genomic information at low cost from real-world samples.

## 2 Results

### 2.1 Comparison of existing assays

**Fig. 1:**
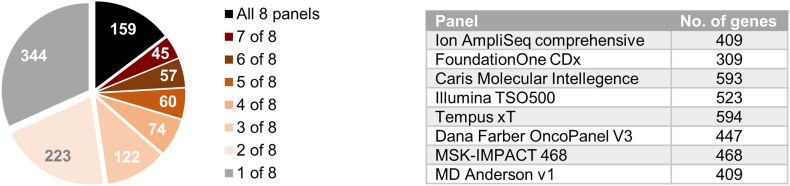
Comparison of cancer NGS assay gene content from commercial and healthcare providers. Piechart displays the intersect of genes across 8 cancer NGS diagnostics (table; total *n* = 1084 genes), showing that less than 15% of genes are covered by all 8 assays, with over half the genes targeted found on only 1 or 2 panels.

A range of sequencing-based cancer diagnostics are currently available from commercial and healthcare providers. These assays commonly report information for 400–500 genes. While this is broadly consistent with estimates of the total number of genes that play a biological role in cancer (i.e. the total number of cancer genes) [15,16,23], analysis of the overlap in content between eight high-profile providers reveals poor correlation, with less than 15% of genes included in all tests, and over half of the genes present in only 1 or 2 assays (Fig. 1). Technical performance aside, this finding highlights significant variability in the quantity and quality of clinically meaningful genomic data that is generated by existing cancer assays.

### 2.2 Defining assay content: Gene level alterations

A list of 2002 candidate cancer genes was generated by combining the outputs of large statistical driver gene studies [[14], [15], [16]] with genes included in commercial and healthcare assays (Fig. 1 and AACR-GENIE v5.0 [1]). The statistical studies are probabilistic in nature and include a false discovery rate (FDR) which is generally set at 5–10%. As a consequence, aggregating outputs from these models leads to an accumulation of false positives (which are different between studies), while intersecting outputs from multiple studies identifies true positives as those that are identified as cancer genes by independent studies. Consistent with this, of the 1474 potential cancer genes identified by the 8 studies of small variant drivers (Supplementary Table 1), only 2.2% of genes were identified by all 8 approaches, and 48.5% of genes were only identified by a single study (Supplementary Fig. 1).

**Fig. 2:**
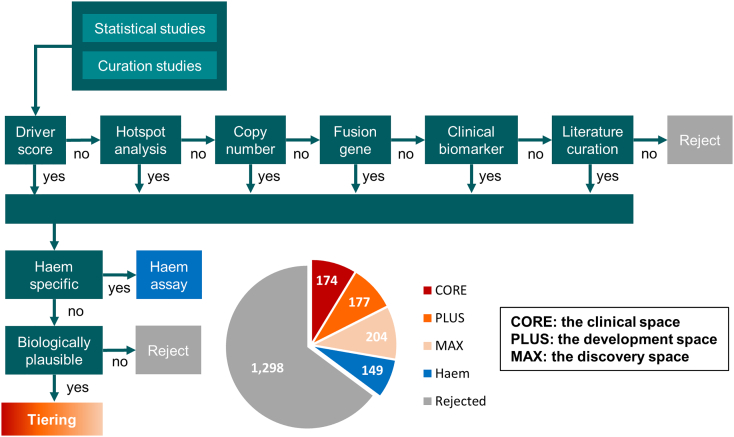
Cancer gene selection decision tree. Schematic of the process used to identify high-confidence cancer driver genes. The pie chart shows the assessment and tiering of 2002 genes present on commercial or LDT-delivered cancer panels.

The 2002 genes were scored and filtered based on their occurrence in nineteen studies (Supplementary Table 1) using mutation, hotspot, copy number and fusion scores (Fig. 2). This approach yielded a set of 447 high-confidence cancer genes. In order to future-proof this gene set, it was augmented with knowledge of biomarkers with potential utility from emerging clinical trial data and/or strong pre-clinical rationale for a role in cancer. Particular attention was paid to genomic markers of response and resistance to treatment, including immunotherapy [12], as genomic alterations resulting in therapy resistance may reside outside of known cancer genes, and could, therefore, be missed by the curation of cancer gene studies. A small number of genes (*n* = 12) that passed the filtering but lacked biological plausibility were excluded, including receptors for low-density lipoprotein and thyroid stimulating hormone. Some low prevalence cancer types are under-represented in large statistical cohorts and additional genes for these were included through curation of the literature. From the total set of starting genes, 149 genes (43 of which passed screening) were found to be altered only in haematological malignancy; these genes were segregated for inclusion in a separate haemato-oncology assay. The final output of this process yielded 555 genes directly implicated in the genesis or evolution of solid adult tumours.

The 555 solid cancer driver genes identified by this process were then assigned to three tiers based on their current utility in clinical practice and drug development. The first tier, termed cancer CORE, includes genes that have an actual role or immediate potential as clinically relevant biomarkers through informing therapy (resistance/response) or prognosis, including emerging biomarkers being tested in clinical trials, particularly novel mechanisms of response and resistance to immunotherapy, or where strong pre-clinical evidence exists. The next tier, cancer PLUS, comprises high confidence cancer genes without current clinical utility, along with potential markers of therapeutic response/resistance for which mechanisms are yet to be fully characterised. The final tier, cancer MAX, comprises probable cancer genes and markers of therapeutic response/resistance for which current data are more speculative (Fig. 2, Supplementary Table 2).

### 2.3 Analysis of existing assays

**Fig. 3:**
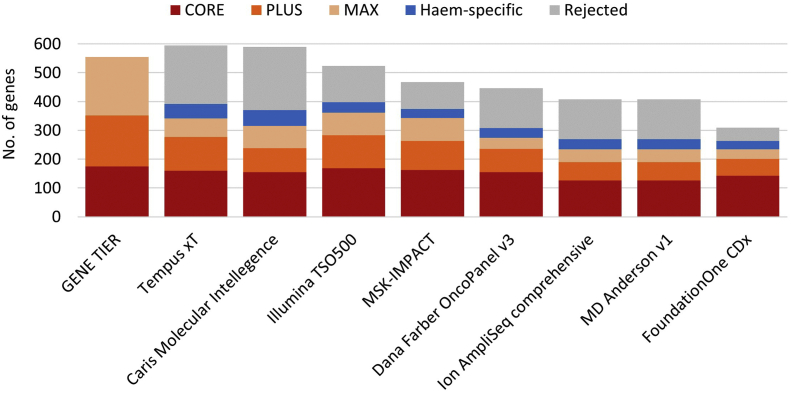
Comparison of cancer NGS assay gene content from commercial and healthcare providers with an objectively defined and clinically tiered set of cancer driver genes.

The tiered list of solid cancer driver genes was mapped back onto the clinical cancer assays analysed in the Fig. 1. This analysis, shown in Fig. 3, demonstrated that clinically important CORE genes were reasonably (but not entirely) represented across the available assays. Notably, many of the assays include a significant proportion of genes for which objective evidence for a role in cancer was not found.

### 2.4 Prevalence of gene-level variant types

Currently available cancer diagnostics have mostly been designed for the detection of small variants (base substitutions and small insertions/deletions). This strategy biases towards the comprehensive characterisation of oncogenes, which are predominantly activated by point mutations, at the expense of tumour suppressor genes, which can be inactivated by mutation, whole or partial gene or exon deletion, or disruptive structural variants (e.g. translocations) within their coding or non-coding footprint. Additional genomic events of relevance include copy number gains and structural variants resulting in fusion genes.

To determine the scope and characteristics of biologically relevant driver genomic alterations targeting the cancer CORE set of genes, we interrogated the PCAWG cohort of high-quality cancer genomes. This cohort of 2348 participants, with unique whole genome cancer sequences representing common solid tumour types, has been analysed by the ICGC PCAWG Consortium to generate a consensus set of genomic aberrations, encompassing all known variant types [16]. As a pre-requisite, oncogene versus tumour suppressor gene (TSG) status was assigned for each gene to facilitate classification of driver versus passenger events. Assignment of TSG versus oncogene status was based on knowledge of both biological function (in normal and malignant states, as well as in therapeutic resistance), and patterns of recurrent mutations observed in cancer. In addition, a third category is required (termed TO) to account for genes that show tumour suppressor or oncogene activity in different tissue contexts, and for genes where different functional states (gain or loss of function) are implicated in oncogenesis and therapeutic response/resistance (for example, *JAK* family genes where activating mutations drive malignant transformation and loss of function mutations impart resistance to immunotherapy) (Supplementary Fig. 2).

Small variants were classed as driver events if they were recurrent in a large data set (AACR-GENIE v5.0) or if they resulted in disruption of genes classified as TSG or TO (see Methods for full details). Gene-level amplification was classified according to consensus criteria for genomic analyses (≥5 copies in a diploid cancer or ≥ 9 copies in a tetraploid cancer; [22,24]). For a subset of oncogenes, a clear link exists between amplification and a role in cancer, including receptor tyrosine kinases and cell cycle components such as cyclin family genes (Supplementary Table 3). For other oncogenes, assigning driver status to copy number gains is not so straightforward, as the biological consequences of amplification are unclear, for example intracellular signalling pathway intermediaries. For these genes, amplification was only annotated as a driver event when amplification was observed in conjunction with a small variant driver. For gene deletion and disruptive structural variants, driver status was only assigned in the presence of biallelic genomic aberrations (i.e. homozygous deletion, or heterozygous deletion + structural variant and so forth). For fusion events, structural variants targeting a subset of oncogenes in the CORE set known to be fusion partners were screened for potential gene fusions and other activating structural variants; rearrangements were considered as drivers if the variant resulted in a known activating event or intersected a reported fusion partner of the oncogene.

**Fig. 4:**
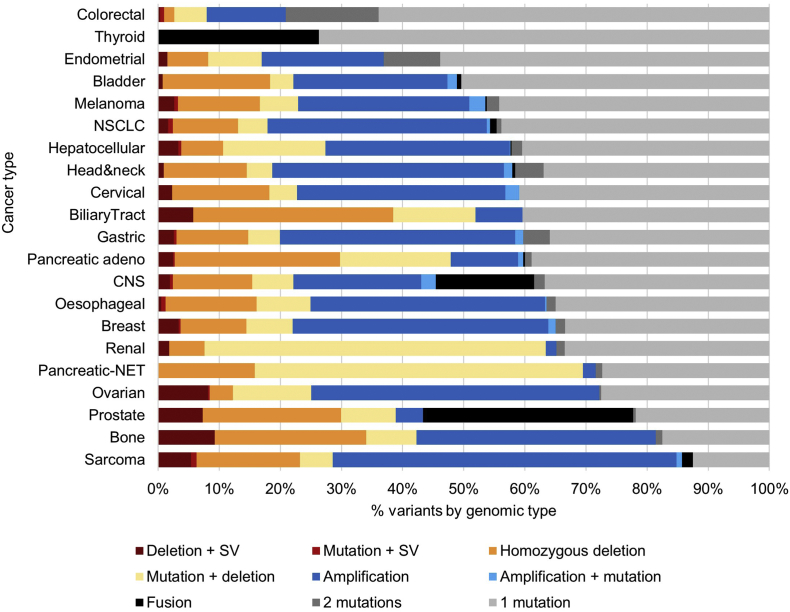
Contribution of different genomic variant types to the important driver events underpinning different cancer types. Analysis of the cancer CORE set of relevant genes. Note: due to the diverse targets, not all of which are covered in this analysis, the contribution of fusions will be under-estimated in the sarcoma profile; no FGFR fusions were detected in the biliary tract samples which is likely due to the lower prevalence of these alterations in East Asia (cohort origin: Singapore). SV: disruptive structural variant; mutation: single nucleotide variants and indels; NET: neuroendocrine tumour. Cancer type [number of samples]: colorectal [52], thyroid [48], endometrial [44], bladder [23], melanoma [107], NSCLC [84], hepatocellular [336], head & neck [56], cervical [20], biliary tract [12], gastric [68], pancreatic adeno [232], CNS [287], oesophageal [97], breast [211], renal [186], pancreatic-NET [81], ovarian [110], prostate [199], bone [61], sarcoma [34].

Integration of cancer CORE driver events in the PCAWG cohort provided an overview of how different variant classes contribute to biologically and clinically relevant events across cancer types (Fig. 4). This analysis highlighted the critical contribution of structural and copy number alterations and verifies previous studies that have identified cancers such as colorectal as largely small variant driven, in contrast to rearrangement-driven tumours such as ovarian cancer and sarcoma [4].

**Fig. 5:**
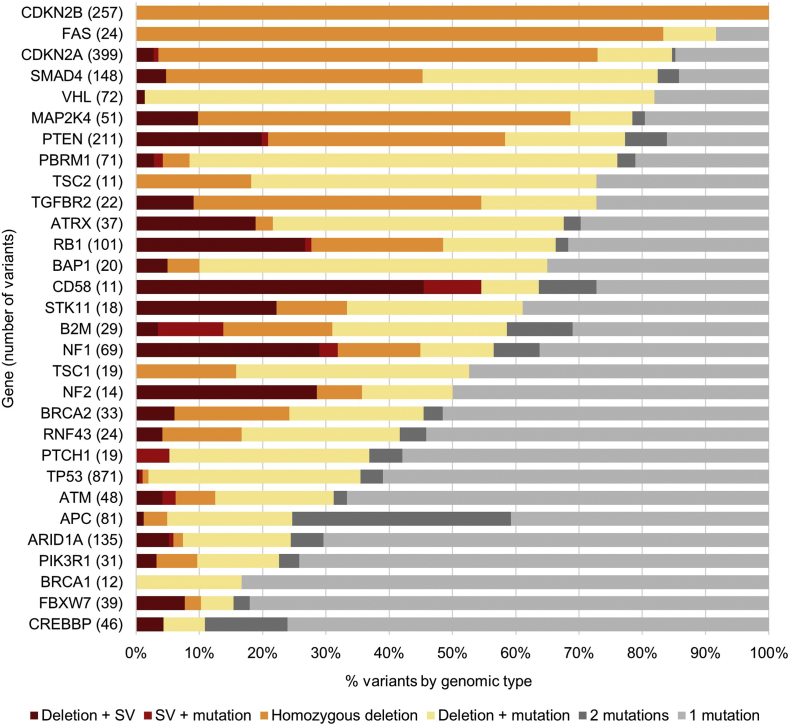
Contribution of different genomic variant combinations to the inactivation of key tumour suppressor genes. Cancer CORE genes containing 10 or more driver variants across the PCAWG cohort. SV: disruptive structural variant; mutation: single nucleotide variants and indels.

**Fig. 6:**
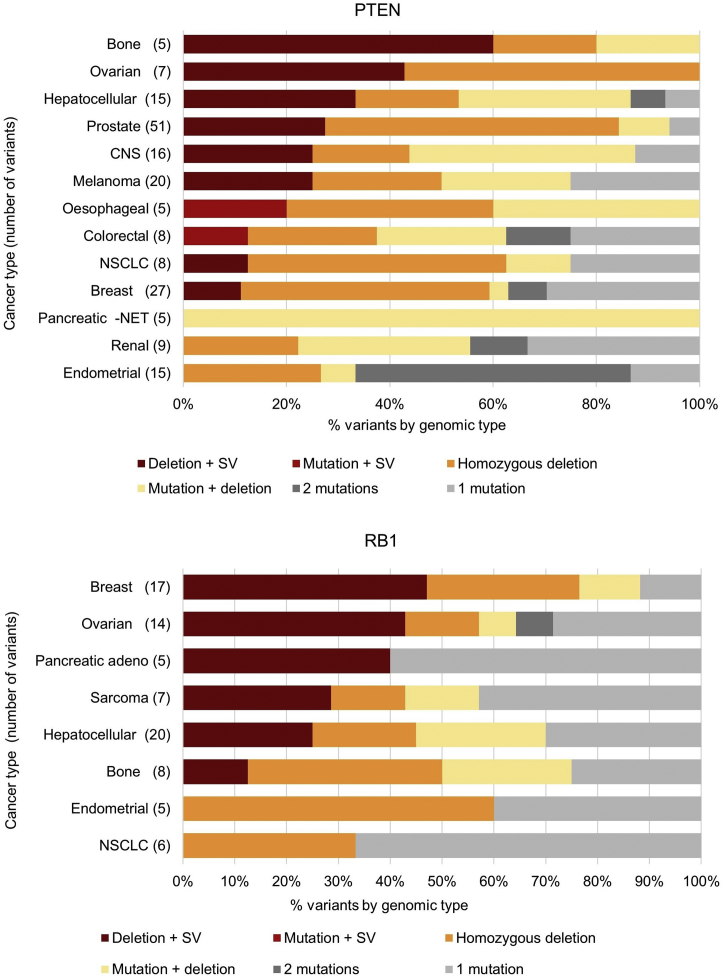
Contribution of different variant classes to genomic events resulting in disruption of PTEN and RB1 tumour suppressor genes. Cancer types with 5 or more driver variants in the relevant gene are shown. SV: disruptive structural variant; mutation: single nucleotide variants and indels; NET: neuroendocrine tumour.

Fig. 4 highlights the important contribution of disruptive structural variants to cancer biology. To further investigate this under-appreciated class of variant, the contribution of different variant types was examined in important tumour suppressor genes (Fig. 5). This demonstrated that the contribution of structural variants to TSG inactivation is highly variable, with inactivation of a subset of TSGs being frequently due to this variant class. Genes commonly inactivated by disruptive structural variants include *CD58* (55%), *NF1* (32%), *RB1* (28%) and *PTEN* (21%). The contribution of structural variants to inactivation of each TSG is also highly variable between tumour types (Fig. 6).

Disruptive structural variants are not detected by the majority of diagnostics currently in clinical use. Failure to detect such events has the potential to adversely impact both clinical decision-making and therapeutic development. Among the tumour suppressor genes commonly targeted by disruptive structural variants, there are a number that are important in current therapeutic development. Loss of PTEN function is associated with hyperactivation of PI3K signalling which can be targeted by small molecule inhibitors, with clinical trials currently underway in prostate cancer [10]. Importantly, 27% of *PTEN* loss in prostate cancer involves a disruptive structural variant (Fig. 6), which would not be detected by currently available diagnostics. Failure to identify the full complement of relevant genomic events risks either depriving patients of a potentially useful therapy or yielding a false-negative clinical trial outcome due to inaccurate patient selection. Another example of a gene commonly inactivated by structural variants is *RB1*, an important biomarker of resistance to CDK4/6 inhibitors such as palbociclib, with this class of event contributing to 47% and 43% of *RB1* inactivation in breast and ovarian cancer respectively (Fig. 6). Together, these findings underscore the importance of the defining and capturing the full range of genomic events that alter the activity of known cancer biomarkers.

## 3 Discussion

**Fig. 7:**
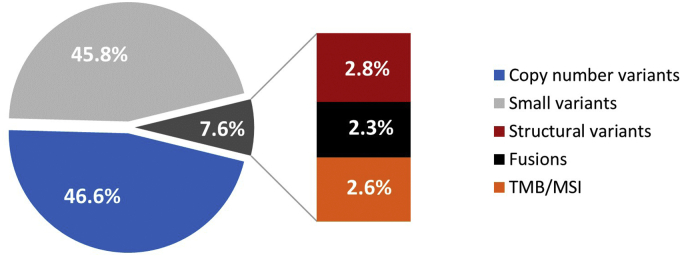
Pan-cancer distribution of cancer relevant genomic information. Proportion of clinically relevant information delivered by variant class across 2348 samples from the PCAWG pan-cancer cohort. TMB: high tumour mutational burden ≥12 mutations/Mb; MSI: microsatellite instability.

Equitable access to genomic testing requires a tumour-type agnostic platform that is both compatible with real-world delivery and maximises the amount of useful genomic information generated. This study provides a pathway to the development of such assays, by defining the extent of the biologically and clinically relevant genomic space, and by characterising the classes and prevalence of genomic events that report this information. A key finding is the necessity to look beyond small coding variants in order to capture clinically useful information (Fig. 7), with disruptive structural variants highlighted as a clinically impactful variant class that is not currently reported by the majority of available assays.

The cancer CORE feature set defines the current clinical biomarker space, covering approved therapy, clinical trials and strong pre-clinical biomarkers, along with markers of prognosis and treatment response/resistance including immunotherapy for adult solid tumours. Targeted capture sequencing provides a technology platform to deliver the vast majority of this information using routinely processed clinical biopsy samples from common adult tumours. The PLUS and the MAX feature sets can be deployed for hypothesis-driven biomarker discovery and agnostic discovery respectively in therapeutic development.

Genes that are specific to haematological cancers were excluded from the gene list, which is intended to be used for the to development of assays for common adult solid tumours. It is important to note, however, that the list of haematology-specific genes identified by this study is unlikely to represent the entirety of the genes implicated in haematological malignancy, as the datasets used in this study are relatively underpowered for the detection of haemato-oncology driver genes. The completion of a haemato-oncology assay would require analysis and curation of additional specific datasets to ensure comprehensive coverage.

Whilst gene-level variants have traditionally been the major focus of cancer research, genome-level alterations provide additional clinically and biologically relevant information. Independent studies have highlighted the potential prognostic impact of the degree of genomic disorganisation within a tumour [19], and microsatellite instability and tumour mutational burden have recently risen to prominence in the context of predicting response to immunotherapy [12]. Additional predictors of response to immunotherapy that lie outside of the coding genome include the presence of viral DNA and the activity of endogenous retrotransposons [13,21].

A well-designed cancer assay is able to capture the vast majority of clinically relevant genomic information through targeted capture and sequencing of DNA, including gene level information as well as the genome-level alterations outlined above. For genes harbouring coding region drivers, targeting of all coding exons, including essential splice sites, is optimal for tumour suppressor genes and preferable for oncogenes. For tumour suppressor genes that are inactivated by structural variants (Fig. 5), targeting of the whole gene footprint is required in order to capture these events. For genes targeted by copy number alterations, assay design should include enough targeted regions to ensure good resolution. This is particularly important for small genes such as B2M and BCL2 which may require additional regional intronic tiling in order to provide sufficient copy number resolution. For genes that are only targeted by copy number alteration and not by small variants, tiling of the entire coding region may not be necessary. The inclusion of a genome-wide copy number backbone can improve the quality of both gene and chromosome level copy number calling. The capture of gene fusions at the DNA level can be limited if the size of the genomic region in which the breakpoints cluster is large. Fortunately, the majority of the clinically important fusions found in common adult solid tumours can be captured at the DNA level, with tiling focused on the introns where the fusion breakpoints fall. Regarding tumour mutational burden, current evidence suggests that a genomic footprint of 1–1.5 Mb is sufficient for accurate estimation. Additional genomic features that may be considered include regions of microsatellite instability, retrotransposons and genotyping SNPs (to ensure sample integrity).

The content and design of individual cancer assays brings together considerations including target cancer type(s), the classes of variants to be detected and the overall size (and thus reagent costs) of the assay. Pan-cancer mutation prevalence for the driver genes identified in this study is shown in Supplementary Table 2, complemented by breakdown by tumour type and by variant class in Supplementary Tables 4 and 5 respectively. Together, this information can be used to prioritise genes for inclusion, and to ensure that assays are designed to capture all relevant classes of genomic variant. Example overviews of potential assay designs are shown in Supplementary Table 6.

The Cancer CORE feature list can be converted into a targeted capture assay with a genomic footprint of approximately 1.8 Mb. This includes full footprint tiling of 12 clinically important tumour suppressor genes and capture of 12 gene fusions, along with genome-wide copy number, microsatellite instability, tumour mutational burden and retrotransposon activity. The reagent costs for this type of design (covering targeted capture library preparation and Illumina sequencing) are in the region of €200 per sample. As such, this represents an affordable assay that is able to deliver the vast majority of clinically relevant information for common adult solid tumours.

Together, this analysis provides a roadmap to unlock the utility of aggregated genomic knowledge, through the delivery of comprehensive genomic profiling for all cancer patients as part of routine clinical care.

## 4 Methods

High-confidence cancer genes were defined according to objective criteria using data garnered from a comprehensive review of published analyses and genomic datasets [[1], [5], [6], [7], [14], [22], [23], [24], [25], [28]] (Supplementary Table 1). For each gene, objective evidence supporting a role for somatic alteration in cancer was compiled by intersecting independent data sources, including studies based on mathematical models, to identify genes enriched for protein-altering mutations (‘driver genes’), genes harbouring recurrently mutated ‘hotspot’ codons, regions of recurrent copy number loss or gain, and recurrent gene fusion events (see Supplementary Methods for full details). The approach is predicated on the notion that each individual approach to driver gene identification will generate both true positive and false positive results. True positive results with be replicated by different studies using either different methodologies or different underlying datasets. False positive results, however, are likely to be dataset and/or analytical method dependent, and are unlikely to be reproduced by different studies. Thus driver events identified by orthogonal approaches will be enriched for true driver genes. The variant types resulting in oncogenic alterations in the subset of these genes with current clinical utility were then characterised across 2348 unique whole genome sequences from solid tumours from the ICGC Pan-Cancer Analysis of Whole Genomes cohort [16], for which analysis of all classes of genomic alteration is available.

## Author contributions

Study conception and design: SLC, PAB, DKC, AVB; data analysis: SLC, PAB; wrote manuscript: PAB, SLC, AVB; attracted funding for the study: AVB, DKC. All main authors read and approved the final manuscript.

## Disclosures

PAB provides consultancy on cancer genomics and drug development for Karus Therapeutics, OncoDNA and Cambridge Cancer Genomics; AVB provides services including consultancy, advisory boards and receives research support from 10.13039/100006436Celgene, AstraZeneca and Elstar Therapeutics; none of these represent a conflict of interest with the content of this publication. All remaining authors have declared no conflicts of interest.

## Appendix A Supplementary data

Supplementary material 1 Image 1

Supplementary material 2 Image 2
