# Supplementary material for: Defining the clinical genomic landscape for real-world precision oncology
Source: Genomics. 2020 Nov;112(6):5324–30. doi: 10.1016/j.ygeno.2020.10.032 (PMC7758710; doi:10.1016/j.ygeno.2020.10.032)
Supplement: Supplementary file 2 — Supplementary material 2 [file mmc2.docx]

**Defining the clinical genomic landscape for real-world precision oncology**

**Supplementary information**

Philip A Beer^1,2^*, Susanna L Cooke^2^*, David K Chang^2,3^ and Andrew V Biankin^2,3,4^.

^1^Sanger Institute, Wellcome Trust Genome Campus, Cambridge CB10 1SA, UNITED KINGDOM; ^2^Wolfson Wohl Cancer Research Centre, Institute of Cancer Sciences, University of Glasgow, Garscube Estate, Switchback Road, Bearsden, Glasgow, Scotland G61 1QH, UNITED KINGDOM; ^3^West of Scotland Pancreatic Unit, Glasgow Royal Infirmary, Glasgow G31 2ER UNITED KINGDOM; ^4^South Western Sydney Clinical School, Goulburn St, Liverpool NSW 2170, AUSTRALIA.

*Equal contribution

**Corresponding author:**

**Andrew V Biankin**

Wolfson Wohl Cancer Research Centre,

Institute of Cancer Sciences, University of Glasgow,

Garscube Estate, Switchback Road,

Bearsden, Glasgow Scotland G61 1BD

Tel: +44 141 330 5670 Fax: +44 141 330 5834

Email: andrew.biankin@glasgow.ac.uk

**SUPPLEMENTARY METHODS**

***Driver gene: small variants***

The cancer genes identified by eight studies (small variant studies, Supplementary Table 1) were intersected to generate a score (0-8) which was applied to a list of potential cancer genes accrued from these studies along with commercial/healthcare delivered assays. Filter pass was defined as a score of ≥3, or a score of 2 where one of the studies was the recent combined informatic analysis of all TCGA samples available to date (Bailey 2018). Genes failing these filters were then queried against an informatic study looking for missense and indel hotspots (Chang 2018) and an in-house curation of hotspots found in the AACR-GENIE dataset. The in-house curated hotspot list was generated from an analysis of the GENE v8.0 release following removal of duplicate samples, artefacts and germline variants; a codon was classed as a mutational hotspot if it harboured ≥10 missense mutations or ≥5 in-frame indels. Any genes harbouring two or more hotspots identified by either of these approaches was included as a driver gene.

***Driver genes: copy number alterations***

Copy number annotation was generated by intersecting two informatic studies of gene level copy number alteration. In the first study (Zack 2013), the output was arrayed to generate a CNV score reporting the number of tumour types in which the gene was deleted or amplified (0-9: CNV score), whether the gene was significantly deleted or amplified in an aggregated analysis (pan-cancer+) and whether the gene was the likely biological target of the copy number segment in which it was located (target+). These data were intersected with the output of a similar gene-level copy number analysis from a second dataset (ICGC-PCAWG). Genes were classed as copy number drivers if one of the following conditions were met, based on the notion that genes with decreasing CNV scores required increasing levels of additional supporting information in order to be classified as driver genes: CNV score ≥7, CNV score 6 plus either pan-cancer+ or PCAWG+, CNV score 4-5 and pan-cancer+ and (either PCAWG+ or target+), CNV score 3 and pan-cancer+ and PCAWG+ and target+. Genes were also included if homozygous loss was identified as a recurrent event in a pan-cancer analysis (Cheng 2017).

***Driver genes: fusion genes***

The top 200 gene hits (by fusion prevalence as number of mutated samples) from six gene fusion datasets (Supplementary Table 1) were intersected to generate a fusion score out of six and a mean prevalence. Genes were classed as fusion drivers if either of the following conditions were met: fusion score ≥3, fusion score 2 and mean prevalence ≥1%.

***Driver genes: emerging biomarkers***

Emerging biomarkers, comprise genes of relevance to drug development or drug resistance, were curated through real-time assessment of published literature and clinical trial horizon scanning.

***Driver mutation annotation: recurrence score***

Small variants were annotated for driver status through recurrence in a large dataset. AACR-GENIE v5.0 was used for this purpose, comprising samples from 56,970 unique patients (duplicate samples from the same patient were removed). Recurrence scores were calculated by codon (i.e. not by alternative allele) for the different small variant types (missense, nonsense, frameshift, in-frame indel). Alleles were excluded if (i) ExAC call ≥10, (ii) allele reported by a single institution using amplicon-based technology, or (iii) ≥90% of the samples for an allele were contributed by institutions using amplicon based technology (steps ii and iii were required to remove artefact from the dataset). Alleles were annotated as drivers if the recurrence score at the codon position was ≥20 for missense variants, and ≥5 for in-frame indels. A small number of curated rare variants for which clear evidence exists for a biological role in cancer were rescued.

***Copy number, structural variant and fusion gene annotation***

Amplification status for ONC and TO genes was calculated based on the total and minor allele counts. Genes were classed as amplified if one of the following conditions were met: (i) total allele ≥5 and minor allele 0 or 1 (diploid background), (ii) total allele ≥9 and minor allele 2 or 3 (triploid or tetraploid background), or (iii) total/minor allele ratio ≥3 and total allele ≥9 (higher ploidy cancers). Gene deletions targeting TSG or TO genes were only included where evidence for biallelic loss of function was present, i.e. homozygous deletion, heterozygous deletion plus driver mutation or heterozygous deletion plus disruptive structural variant. Similarly, disruptive structural variants (SV) targeting TSG or TO genes were only included where evidence for biallelic loss of function was present, i.e. SV plus heterozygous deletion or SV plus driver mutation. SVs comprise any of the following events falling within the coding footprint of the gene: inversion (head-to-head or tail-to-tail), translocation, deletion or duplication (all deletion/duplication events <0.1Mb were inspected visually and events falling entirely within an intron were removed). Disruptive SVs observed in the PCAWG cohort comprise genomic alterations of a range of different sizes, from single exon deletions to large inversions, as well as disruptive translocations.

Copy number variants and SVs are manifestations of the same basic class of genomic alteration. Variants are called as copy number changes where enough of the gene footprint is at an altered state, and in general an associated SV is also detected. Where a deletion and an SV were reported for the same gene, care was taken to distinguish independent events from a single alteration: copy number steps were matched to SV breakpoints (both of which are available at base-pair resolution for the PCAWG cohort) allowing for single alterations to be assigned to one variant class only. Where a deletion was reported as both a copy number variant and an SV, it was annotated by default as a CNV.

The following genes were screened for fusion events: ALK, BRAF, EGFR, ETV6, FGFR2, FGFR3, NTRK1, RAF1, RET, ROS1. Structural variants targeting these genes where no amplification call was made were screened for gene fusion events; variants were annotated as drivers where the structural variant either intersected a reported fusion partner of the oncogene, or in the case of EGFR resulted in a known activating alteration (EGFR vIII or kinase domain duplication). Due to the focused nature of the fusion gene assessment (restricted to genes in the CORE gene set), the fusion gene prevalence for sarcoma will be an under-estimate due to the large number of diverse events present in this cancer type.

**Supplementary Table 1. Datasets and resources informing the identification of cancer genes.**

| **Dataset** | **Analysis** | **Output** | **Reference** |
| --- | --- | --- | --- |
| Kandoth 2013 | Informatic | Small variants | PMID: 24132290 |
| Lawrence 2014 | Informatic | Small variants | PMID: 24390350 |
| Bailey 2018 | Informatic | Small variants | PMID: 30096302 |
| IntOGen | Informatic | Small variants | https://www.intogen.org |
| Vogelstein 2013 | Informatic | Small variants | PMID: 23539594 |
| Database of Curated Mutations (DoCM) | Curated | Small variants | http://docm.info/ |
| The Cancer Gene Census (CGS) | Curated | Small variants | https://cancer.sanger.ac.uk/census |
| OncoKB | Curated | Small variants | PMID: 28890946 |
| Chang 2018 | Informatic | Hotspots | PMID: 29247016 |
| AACR-GENIE (v8.0) | Curated | Hotspots | PMID: 28572459 |
| Zack 2013 | Informatic | CNVs | PMID: 24071852 |
| Cheng 2017 | Informatic | CNVs | PMID: 29089486 |
| PCAWG | Informatic | CNVs | https://docs.icgc.org/pcawg/ |
| Yoshihara 2015 | Informatic | Gene fusions | PMID: 25500544 |
| Gao 2018 | Informatic | Gene fusions | PMID: 29617662 |
| Atlas of Genetics and Cytogenetics | Curated | Gene fusions | http://atlasgeneticsoncology.org/ |
| COSMIC | Curated | Gene fusions | https://cancer.sanger.ac.uk/cosmic |
| AACR GENIE (v5) | Dataset | Gene fusions | PMID: 28572459 |
| The Cancer Genome Atlas | Dataset | Gene fusions | http://www.cbioportal.org/ |

*CNV: copy number variant; AACR GENIE: American Association for Cancer Research Genomics Evidence Neoplasia Information Exchange*


**Supplementary Table 3. Oncogenes activated by amplification.**

| MDM2 | KIT | CDK2 | FGFR4 |
| --- | --- | --- | --- |
| EGFR | CDK6 | MYC | MCL1 |
| CDK4 | IGF1R | FGFR2 | PDGFRB |
| CCND1 | HGF | CCND3 | MAX |
| FGF19 | MYCN | MET | BCL2 |
| ERBB2 | ERBB3 | FGFR3 | SYK |
| CCNE1 | CCND2 | SRC | ERBB4 |
| YAP1 | FGFR1 | KDR | JAK3 |
| PDGFRA | MYB | AURKA | JAK2 |
| JUN | TERT | AXL | JAK1 |
